# Cell-Type Specific Deletion of CB2 Cannabinoid Receptors in Dopamine Neurons Induced Hyperactivity Phenotype: Possible Relevance to Attention-Deficit Hyperactivity Disorder

**DOI:** 10.3389/fpsyt.2021.803394

**Published:** 2022-02-08

**Authors:** Ana Canseco-Alba, Branden Sanabria, Mariam Hammouda, Rollanda Bernadin, Marizel Mina, Qing-Rong Liu, Emmanuel S. Onaivi

**Affiliations:** ^1^Dirección de Investigación, Instituto Nacional de Neurología y Neurocirugía “Manuel Velasco Suárez”, Mexico City, Mexico; ^2^Department of Biology, William Paterson University, Wayne, NJ, United States; ^3^Laboratory of Clinical Investigation, National Institute on Aging, National Institutes of Health, Baltimore, MD, United States

**Keywords:** endocannabinoid system, dopamine, adolescence, locomotor activity, amphetamine

## Abstract

DAT-*Cnr2* mice are conditional knockout (cKO) animals that do not express cannabinoid CB2 receptors (CB2R), in midbrain dopamine neurons. The hyperactivity phenotype of DAT-*Cnr2* cKO mice were paradoxically reduced by low dose of amphetamine. Here, we report on the locomotor activity analysis in male and female adolescent (PND 30 ± 2) mice in basal conditions and in response to different doses of amphetamine, using the Open Field (OF), Elevated Plus-Maze (EPM) tests and the Novel Object Recognition (NOR) task as a putative model of attention deficit hyperactivity disorder (ADHD). Results showed that both male and female adolescent DAT-*Cnr2* mice displayed significant increases in distance traveled in the OF test compared with WT mice. However, 2 mg/kg dose of amphetamine reduced the distance traveled by the DAT-*Cnr2* but was increased in the WT mice. In the EPM test of anxiety-like behavioral responses, DAT-*Cnr2* spent more time in the open arms of the maze than the WT mice, suggesting a reduction in anxiety-like response. DAT-*Cnr2* mice showed significant increase in the number of unprotected head dips in the maze test and in the cliff avoidance reaction (CAR) test demonstrating impulsivity and risky behavior. DAT-*Cnr2* mice also exhibited deficient response in the delay decision making (DDM), with impulsive choice. Both DAT-*Cnr2* and WT were able to recognize the new object in the NOR task, but the exploration by the DAT-*Cnr2* was less than that of the WT mice. Following the administration of 2 mg/kg of amphetamine, the similarities and differential performances of the DAT-*Cnr2* and WT mice in the EPM test and NOR task was probably due to increase in attention. Microglia activation detected by Cd11b immunolabelling was enhanced in the hippocampus in DAT*-Cnr2* cKO than in WT mice, implicating neuro-immune modulatory effects of CB2R. The results demonstrates that DAT-*Cnr2* cKO mice with cell-type specific deletion of CB2R in midbrain dopaminergic neurons may represent a possible model for studying the neurobiological basis of ADHD.

## Introduction

The study of cannabinoid compounds, such as Δ^9^-THC, the psychoactive molecule in *Cannabis sativa*, led to the discovery first of specific receptors and then to a new system in the mammalian body: the endocannabinoid system (ECS) (1). The ECS is composed of two receptor subtypes: CB1R, CB2R, their endogenous ligands endocannabinoids (eCBs), the enzymes for the synthesis and degradation of endocannabinoids, and the reuptake transport system (2). There are new insights to an expanded ECS—the endocannabinoidome (eCBome). The eCBome is widely distributed throughout the body (3, 4). In the brain, the ECS is a lipid signaling system, which is functionally active at the early stages of brain development, and plays a neuromodulatory role in several behaviors (3, 5, 6). A functional role of the ECS in neurological and psychiatric disorders has been implicated, for example: Parkinson's disease, anxiety, depression, schizophrenia, attention deficit hyperactivity disorder (ADHD), among others (7, 8).

Despite the fact that both CB1 and CB2 receptors belong to the group of class A G protein-coupled receptors and are characterized by significant homology (44% of their molecular structure), and encoded by different genes, they differ in their function and specificity of cellular expression as well as their pattern of distribution (4, 8, 9). CB1R is highly expressed in the brain and its role on different behaviors is well-documented (3). CB2R is found predominantly in immune cells and have been referred to as peripheral CBRs. However, recent evidence demonstrate that CB2Rs are expressed in microglia and also in neurons in the brain (10–15). The role of CB2R in immune system function has been widely described (16) as well as immune function of CB2R in the brain (17, 18). There is however a growing and exciting research interest involving CB2R in the modulation of neuronal function and behavior (12, 19) and its possible therapeutic implications (20). One of the strategies for the study of CB1R, and the role of receptors in general is the use of knockout mice (21, 22). CB2Rs in the brain are expressed in postsynaptic somatodendritic region of neurons in discrete areas, at much lower levels than the CB1R (15). Hence, the use of technologies such as Cre-loxP strategies are crucial, since it is possible to study the participation of the CB2R on specific behaviors induced by particular brain areas (12, 23). One of the areas where CB2R is expressed in the brain is on the dopaminergic neurons of the midbrain (13). The cell bodies of midbrain dopamine neurons in groups A8 (dorsal to lateral substantia nigra), A9 (pars compacta of substantia nigra) and A10 (ventral tegmental area medial to substantia nigra) are located in the midbrain. These neurons express DA transporters (DAT) specifically and are critical for controlling voluntary movement, creating associations with rewarding stimuli, attending to salient environmental stimuli, motivating behavior, maintenance of working memory and the regulation of emotion (24). Its dysregulation is implicated across many neurological and psychiatric disorders, such as Parkinson's disease (25), schizophrenia (26, 27), addiction (28) and attention-deficit hyperactivity disorder (ADHD) (29–31).

DAT-*Cnr2* mice are conditional knockout (cKO) mice with specific deletion of CB2R in dopamine neurons, which allows us to demonstrate that CB2R are implicated with the regulation of locomotor activity and in the modulation of dopaminergic transmission. This is because, the deletion of the CB2R in DA neurons release a “brake” on psychomotor activity resulting in continuous spontaneous hyperactivity (32). This hyperactivity was consistently found in all the tests performed (32). Hyperactivity, along with inattentiveness and impulsivity are core behavioral symptoms of ADHD, according to the Diagnostic and Statistical Manual of Mental Disorders (DSM-5) (33). While the mechanism(s) and cause of ADHD is incompletely understood, it is a debilitating disorder that disrupts routine functioning and/or development. In previous study with DAT-*Cnr2* mice, we characterized the effects of different psychostimulants on locomotion, the induction of sensitization and their rewarding properties. One of the more intriguing findings was that the effects of a low dose of amphetamine (2 mg/kg) reduced locomotor activity of the DAT-*Cnr2* cKO adult male mice, which is contrary to the effect of the same dose in paired WT mice as control group. In addition, DAT-*Cnr2* cKO mice overexpressed the enzyme tyrosine hydroxylase (TH) suggesting a hyper-dopaminergic phenotype (34). This paradoxical effect, the reduction of hyperactivity in response to amphetamine, and the intrinsic hyperactivity, are considered as criteria for ADHD models (35). The most common pharmacological treatment prescribed for ADHD is the administration of amphetamine or methylphenidate (36, 37). Furthermore, imbalances in dopaminergic and noradrenergic systems have been implicated in the core symptoms that characterize this disorder (38, 39), although the neurobiology of ADHD is not completely understood. These two key findings, the hyperactivity and the paradoxical effect to amphetamine, motivated the characterization of DAT-*Cnr2* cKO adolescent mice, since all the previous data was obtained from adults. The objective of this work was to evaluate locomotor activity of DAT-*Cnr2* cKO adolescent mice in basal conditions and in response to different doses of amphetamine. To evaluate the performance of adolescent mice in the anxiety-like behavioral test, the elevated plus-maze (EPM) paradigm was utilized. We previously demonstrated that adult DAT-*Cnr2* mice display less anxiety in the EPM and other anxiety-like tests. However, we were interested not only in the time spent in the open-close arms, which is indicative of anxiety-like behavior, but in the number of unprotected head dips, as a measure of risky behavior and impulsivity. For a better understanding of the possible differences in risky behavior and impulsivity in the DAT-*Cnr2* mice, we assessed maladaptive impulsive rodent behavior by the cliff avoidance reaction (CAR) paradigm (40), and impulsive choice by a delay- and effort-based decision-making (DDM & EDM) T-maze test. It is considered to be a behavioral measure of impulsivity since it assesses impulsive decision making. Finally, we were interested in the performance of adolescent DAT-*Cnr2* mice not only in the novel object recognition task (NOR) and cognitive deficits (learning and memory), but also to determine their explorative behavior, as an indirect indicator of attention. The performance of DAT-*Cnr2* mice in EPM and NOR in response to a single administration of a low dose of amphetamine was also investigated. Coronal sections of dentate gyrus and cornu ammonis (CA) from DAT-*Cnr2* and WT mice were stained with Cd11b, a marker for microglia activation for the neuro-immuno modulating effects of CB2R. Thus, the hypothesis that DAT-*Cnr2* cKO mice may be a valid model for studying attention deficit hyperactivity disorder (ADHD) was tested.

## Materials and Methods

### Animals

DAT-*Cnr*2^−/−^ male and female adolescent mice (PND 30 ± 2) and C57BL/6J male and female (PND 30 ± 2) mice as wild type (WT) were used in this study. The genotypes of the cKO mice were carried out by TransnetYX (Cordova, TN). All mice were housed in groups of four in acrylic home cages (25 × 25 × 14.5 cm). To reduce their stress levels the mice were handled daily before the initiation of the experiments. All mice were housed under the following conditions: constant temperature; a reversed light schedule (dim red light on 19:30–07:30 h); and food and water freely available. This study was approved by the Institutional Animal Care and Use Committee (IACUC) of the William Paterson University. The details on the generation of the conditional knock out mice has been described elsewhere (32).

### Drugs

The psychostimulant (+)-amphetamine sulfate (amphetamine) was dissolved in 0.9% saline NaCl) and administered into the peritoneum (i.p.) at a volume of 0.01 ml/g body weight. The drug was purchased from Sigma-Aldrich Chem. Co. (St. Louis, Mo, USA). The doses tested were: 0.1, 2.0, and 5.0 mg/kg. The experiments started 15 min after the injection. The vehicle (saline) was given to the control animals in the same volume.

### Behavioral Testing

Behavioral performance of the DAT-*Cnr2* cKO and WT mice were evaluated in the open field (OF) test for locomotor activity, in the elevated-plus maze (EPM) test of anxiety-like behavior, in the novel object recognition (NOR) task for measurements of cognitive performance. In an elevated platform for the evaluation of the cliff avoidance reaction (CAR) and in Delay- and Effort-based Decision Making using a T-maze test. All the experiments were performed in a behavioral room under a red dim light during the dark phase of the dark/light cycle. During the day of the experiments, the animals were placed in the room for an hour for habituation and all the behavioral test apparatuses were cleaned after each test using 70% ethanol. In addition to the habituation and handling, mice were moved to the behavioral testing room for an hour before any behavioral experiment was conducted. This is a longer time period than the one used in other protocols. However, the adolescent mice took longer to habituate in pilot studies, so we increased the amount of time in order to habituate them to the different conditions and handling. All the behavioral sessions were video-recorded without the presence of the experimenter to avoid distractions and double-blindly analyzed.

#### Locomotor Activity—Open Field Test

**Figure 1:**
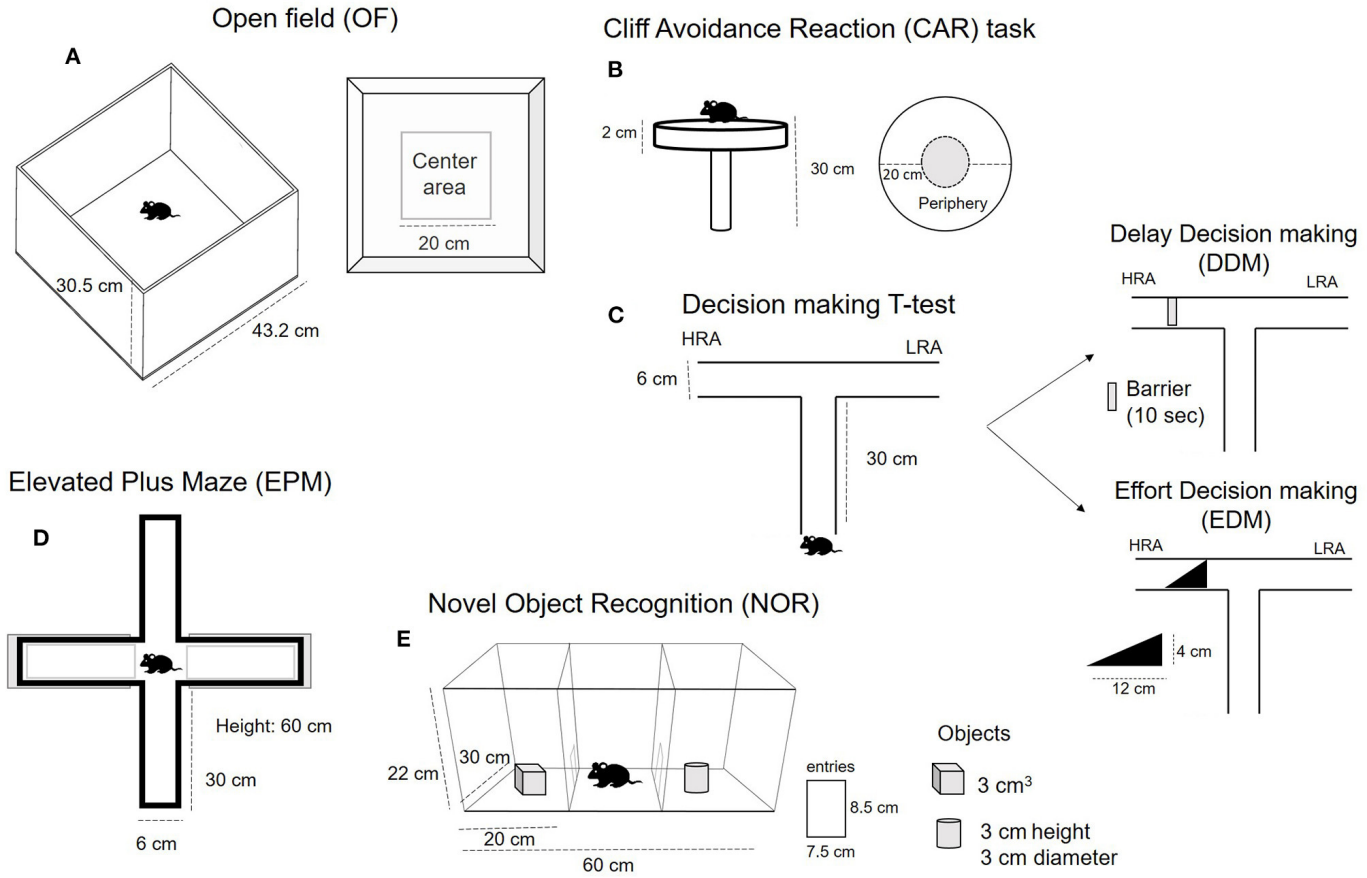
Schematic representation of the behavioral tests used to characterize specific behavior in adolescent male and female DAT-*Cnr2* cKO mice in comparison with WT mice.

To evaluate spontaneous locomotor activity, mice were individually placed into the center of an infrared photobeam-controlled open-field test chamber (43.2 × 43.2 × 30.5 cm; ENV −510: MED Associates Inc., St. Albans, VT, USA) and allowed to freely explore the chamber for 30 min. The test boxes were connected to a computer, and total distance traveled, the number of rearing and stereotypic counts were obtained. The time spent in the center of the open field was also recorded. In order to evaluate habituation to the environment, the distance traveled was compared throughout the course of 3 consecutive days at the exact same time. Repeated exposure provided a method for assessing habituation to the increasingly familiar chamber environment. More details can be found in Figure 1A.

#### Cliff Avoidance Reaction (CAR)

CAR refers to natural tendency of animals to avoid a potential fall from a height. For this test, each mouse was gently placed on a platform so that the forelimbs approached the edge and they were allowed to move freely for a minute. The platform to assess CAR consisted of a round plexiglass (diameter, 20 cm; thickness, 2 cm) supported by a glass rod (height, 30 cm). The platform was secured so that the movement of the animal did not affect it. The floor below the platform was cushioned to prevent injury if the animal fell. We measured the percentage of animals falling from the platform. Since this was an infrequent occurrence, we did not count the number of falls. We determined the time spent exploring the periphery of the elevated platform, the time in the surrounding area as a risky behavior. We recorded the frequency of head dips (we considered a head dip to be when the whole head moved downward at the edge of the platform) and the accumulative time spent with the head dipped. The protocol was similar to those used by other authors (40, 41). Details and scheme can be found in Figure 1B.

#### Delay- and Effort-Based Decision Making (DDM and EDM)

This cost-benefit conflict task allows evaluation of decision making between the attainment of a high reward (HR) over a low reward (LR) (i.e., animals balance goal achievement and effort management) in a T-maze. The mice were well-trained in the maze to obtain a HR on one arm and a LR on the other. The test consisted of a barrier on the HR arm to evaluate if the animal is capable of waiting to obtain the large/delay reward (HR) over the small/immediate reward (LR). This is the Delay based Decision Making (DDM). The other test consisted of the placement of a ramp, so that the animals needed to climb in order to obtain the large reward (HR), which is the Effort-based Decision Making (EDM). This test was used to evaluate impulsivity (42). And the protocol was similar to the one used by other investigators (43, 44). The apparatus consisted of a wooden T-maze (30 cm length of each arm). The vertical arm is neutral and has a door to restrain the mouse before the decision had to be made. The arm with the HR had a removable barrier for the DDM and a ramp (4 cm height; 12 cm length) for the EDM. The test consisted of two phases, the training phase and the test phase. The pre-training lasted 1 day and all the mice were habituated to the experiment and to the T-maze apparatus. Mice were simply allowed to explore the T-maze for 10 min each. No rewards or stimuli were present at this time.

The training phase consisted in conditioning animals to associate one arm with the HR (pellets covered with peanut butter) and the other with the LR (standard pellets). In order to do so, first the LR and HR pellets were placed everywhere in the maze and each mouse was placed in the maze for 10 min to explore. Then, the LR and HR pellets were assigned to the left and right arm, respectively, and the animals were allowed to explore for 10 min three times per day for a period of 3 days. We tested the association by restraining the mouse in one arm, the neutral one, and observed if they preferred the HR arm. By the end of the training, all the animals immediately decided to go to the HR arm when released from the neutral arm. The next day was testing (or evaluation) day. The animals were randomly assigned to the DDM or EDM. For the DDM, the HR arm was blocked by a barrier for 10 s after which the animal was released from the neutral arm. In this case, we measured the percentage of animals choosing the HR or the LR arm, and the time spent close to the barrier and in each arm. We also measured the number of entries into the arms for 3 min. For the EDM, we placed the ramp in front of the HR. In this case, we measured the percentage of animals climbing the ramp to access the HR and the latency and number of entries into the arms before climbing. Schematic representation is shown Figure 1C.

#### Anxiety-Like Behavior—Elevated Plus-Maze (EPM) Test

For this test, each mouse was placed on the central platform of the maze, facing one of the open arms, and allowed to move freely for 5 min. The maze consisted of a plus-shaped maze with two open and two close arms (each arm length 30 cm; arm width 6 cm), each with an open roof, elevated 60 cm from the floor. The maze was cleaned with alcohol between each animal. The time spent in the open and closed arms was recorded for each animal, as well as the number of entries into the arms and the frequency of unprotected head dips as risk assessment behaviors was also monitored. More details can be found in Figure 1D.

#### Cognition—Novel Object Recognition Task (NOR)

This test consisted of two phases: a training phase and a testing phase. In order to reduce the anxiety produced by the new environment, mice were habituated to the arena for 30 min for 4 days before the evaluation, this is a modification from the original protocol since adolescent mice and DAT-*Cnr2* cKO mice in particular are hyperactive and take longer to habituate to the apparatus. The apparatus consisted of a three-chambered arena (30 cm length × 60 cm width × 22 cm height total). An entrance (8.5 cm height and 7.5 width) connected the three chambers and a retractable door. On test days, each mouse was placed in the middle chamber with the door closed for 2 min. Immediately after, the door was opened and the animal was able to move into the arena freely and explore the two objects that were placed into each side chamber for 5 min. In this first phase (training phase), the objects were identical. Next, the animal was placed into its home cage. After 30 min, the animal was placed into the arena, again in the middle chamber for 2 min. When the doors are opened and the animals are free to explore the objects for 5 min, but in this case, one of the objects will be different. The object used were selected to be different enough to be easily discriminated by the mice, but with similar degree of complexity in a size allowing animals to climb on it. A wooden cube and a cylinder (3 cm) were used. The object exploration time was recorded for each phase, as well as the number of entries among the chambers. The NOR test is a model for learning and episodic memory, which evaluated the capacity of the animal to recognize the new object, i.e., increased the time exploring the new one in relation with the old (already known) one. We obtained a discriminatory index using the formular: time exploring the new object divided by the sum of the time exploring the new one plus the old one in the test trial. If the resulting index is higher than 0.5, it means that the animal spent more time exploring the new object. Details are in Figure 1E.

### Histological Assessment

#### Tissue Sample Preparation

Mice were anesthetized with a ketamine/xylazine solution and transcardially perfused with 0.9% NaCl followed by 4.0% paraformaldehyde (PFA) in 0.1M phosphate buffered saline pH 7.4 (PBS). Brains were removed, post-fixed in the same fixative solution overnight and then cryoprotected in 20% sucrose in 4% PFA in PBS. Serial 30 um-thick coronal sections of the hippocampus were obtained from each brain and collected in PBS.

#### Immunohistochemistry (IHC)

Hippocampal sections containing dentate gyrus and *cornu ammonis* (CA) were processed free floating in blocking buffer (5% normal donkey serum, 0.4% Triton X-100, 1% BSA, in 0.01M PBS) for 1 h at room temperature and then incubated with the primary antibody goat anti-CD11b (Abcam, ab62817) at 1:500 for 48 h at 4°C. Sections were washed with PBS then incubated in secondary antibody donkey anti-goat alexa fluor 488 (ab150129) 1:500 for 2 h at RT. Sections were rinsed, counter stained with DAPI (invitrogen, D1306), then mounted onto slides and cover slipped. Immunofluorescence was captured using a Zeiss LSM 700 confocal laser scanning confocal microscope with Zen software and Axio imager (Carl Zeiss). Images were analyzed and were subsequently exported to Tiff or JPEG format.

### Experimental Design

The study can be broadly divided into the measurement of behavior in basal conditions and in response to amphetamine. For the behavioral testing during basal conditions, we randomly assign adolescent male and females of the WT (C57BL/6J) and DAT-*Cnr2* cKO mice in independent groups of 10 and are then subjected to the tests: Open Field (OF), Cliff Avoidance Reaction (CAR) test and Delay- and Effort Decision Making (DDM and EDM). For the evaluation of the effects of amphetamine, first we perform a dose-response curve using eight independent (*n* = 10 each) of each sex, randomly assigned to the following groups: Saline, Amphetamine 0.1 mg/kg, Amphetamine 2.0 mg/kg and Amphetamine 5.0 mg/kg. The animals received acute injection and 15 min later, are placed individually into the activity monitor and then their locomotor activity was recorded for 30 min. The next set of experiments consisted of the evaluation of the effects of 2.0 mg amphetamine in the Elevated Plus Maze (EPM) and in the Novel Recognition Test (NOR). Four independent groups (*n* = 10 each) of each sex were used for each test. The animals were randomly assigned to the Saline or Amphetamine.

In order to determine a possible crosstalk between neurons and immune cells in basal conditions, a separate cohort of five undisturbed DAT-*Cnr2* cKO and five WT mice were used for measurement of the constitutive marker of microglia Cd11b by immuno-labeling in the hippocampus.

### Statistical Analysis

Two-Way ANOVA follow by Tukey Test as *post hoc* was used for the analysis of all the parameters of the open field test (distance traveled, stereotypic counts, rearing count and the time spent in the center of the field). The parameters of the Cliff avoidance reaction (number of head dips, time spent with head dip and time spent in the periphery) was analyzed by Two-Way ANOVA follow by Tukey Test as *post hoc*. The factors were: genotype (WT and DAT-*Cnr2*) and sex (male and female). The comparison of the proportions of the four independent groups was conducted by means of the Fisher F test. For the DDM and EDM, the percentages were compared by the Fisher F test, and for the temporal parameters, a Two-Way ANOVA was performed. Two-Way Repeated Measures Analysis of Variance (RM ANOVA) was used to compare the distance traveled over 3 consecutive days for both genotypes: WT and DAT-*Cnr2* cKO, one analysis for male and another for female mice. Data of the parameters in EPM and NOR tests were analyzed with a three-way ANOVA with “genotype,” “treatment,” and “sex” as factors. *Post-hoc* comparisons were performed with Tukey Test. A level of confidence of *P* < 0.05 was considered statistically significant.

## Results

### Performance in the Open Field Test and Habituation of the Male and Female Adolescent Mice to a New Environment

**Figure 2:**
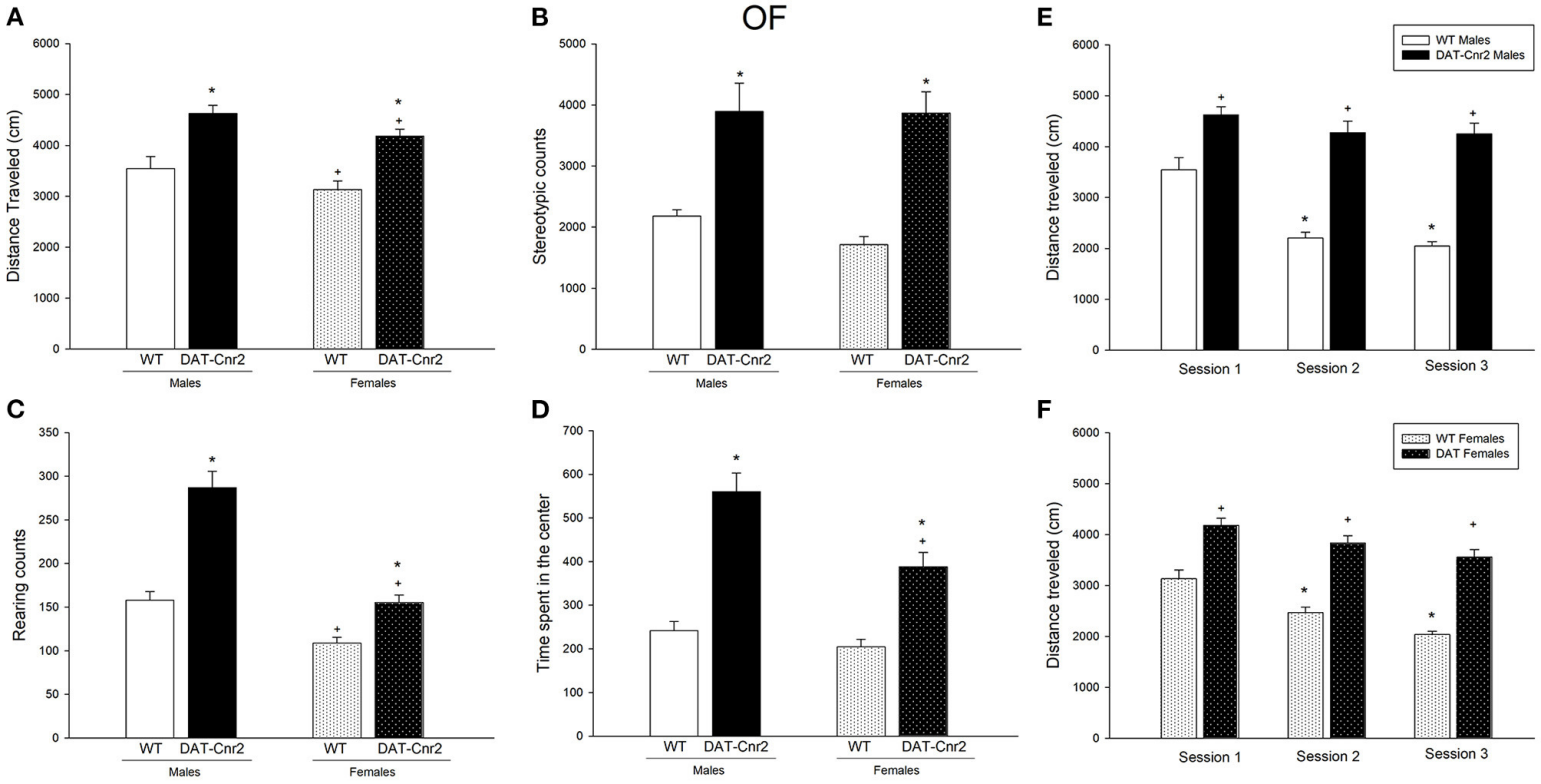
Performance in the Open Field (OF) test in adolescent male and female DAT-*Cnr2* cKO mice. Distance traveled **(A)**, stereotypic counts **(B)**, rearing counts **(C)**, and time spent in the center of the arena **(D)**. Distance traveled during 30 min in the open field in basal conditions for 3 consecutive days, **(E)** for males and **(F)** for females. The white bars represent the WT and the black bars represent DAT-*Cnr2* cKO mice. Data are expressed as mean ± SEM (*n* = 10 mice per group). Two-way ANOVA followed by Tukey *p < 0.05 for genotypes; ^+^*p* < 0.05 for sex and Two way RM ANOVA followed by Tukey for **(E,F)**.

A two-way ANOVA with genotype (WT/DAT-*Cnr2*) as one factor and sex (male/female) as the other factor revealed significant differences in the distance traveled parameter [*F*_(1, 36)_ = 35.169, *p* < 0.001 for genotype and *F*_(1, 36)_ = 5.690, *p* = 0.02 for sex]. *Post hoc* test showed that the distance traveled in the test was significantly higher for the DAT-*Cnr2* adolescent mice in comparison with WT mice. The distance traveled by the females was slightly but significantly lower than the one shown by the males in both genotypes, as it can be seen in Figure 2A. For the stereotypic counts, Figure 2B, there was a significant difference among genotypes [*F*_(2, 36)_ = 40.914, *p* < 0.001], with the DAT-*Cnr2* adolescent mice showing more counts. For the rearing counts, Figure 2C, the analysis revealed significant difference [*F*_(1, 36)_ = 54.035, *p* < 0.001 for genotype; *F*_(1, 36)_ = 57.285, *p* < 0.001 for sex and *F*_(1, 36)_ = 11.825, *p* = 0.001]. The DAT-*Cnr2* cKO adolescent mice are the ones rearing more frequently, followed by the DAT-*Cnr2* adolescent females. Finally, as it can be seen in Figure 2D, the time spent in the center of the open field, considered to be an indicator of anxiety-like behavior revealed significant differences [*F*_(1, 36)_ = 45.264, *p* < 0.001 for genotype; *F*_(1, 36)_ = 26.725, *p* < 0.001 for sex and *F*_(1, 36)_ = 15.385, *p* < 0.001]. DAT-*Cnr2* male adolescent mice spent significantly more time in the center of the arena in comparison with WT adolescent male mice. In females, the time spent in the center for DAT-*Cnr2* was higher than the WT female mice, but did not reach the time spent by the DAT-*Cnr2* mice. Two-Way Repeated Measures Analysis of Variance (RM ANOVA) revealed that the distance traveled by the adolescent WT male mice in the Open Field (OF) decreased after each respective session (3 consecutive days), reflecting habituation to a new environment. In contrast, male DAT-*Cnr2* mice did not show a reduction in the distance traveled at any time. Therefore, the distance traveled was significantly higher in the DAT-*Cnr2* mice in comparison with the WT male mice [*F*_(1, 36)_ = 14.893, *p* < 0.001 for sessions; *F*_(1, 18)_ = 123.245, *p* < 0.001 for genotype]. The same pattern was observed in adolescent female mice [*F*_(2, 18)_ = 47.658, *p* < 0.001 for genotype; *F*_(1, 18)_ = 49.470, *p* < 0.001 for genotype]. Figures 2E,F, respectively.

### Performance in the CAR Test of the Male and Female Adolescent Mice

**Figure 3:**
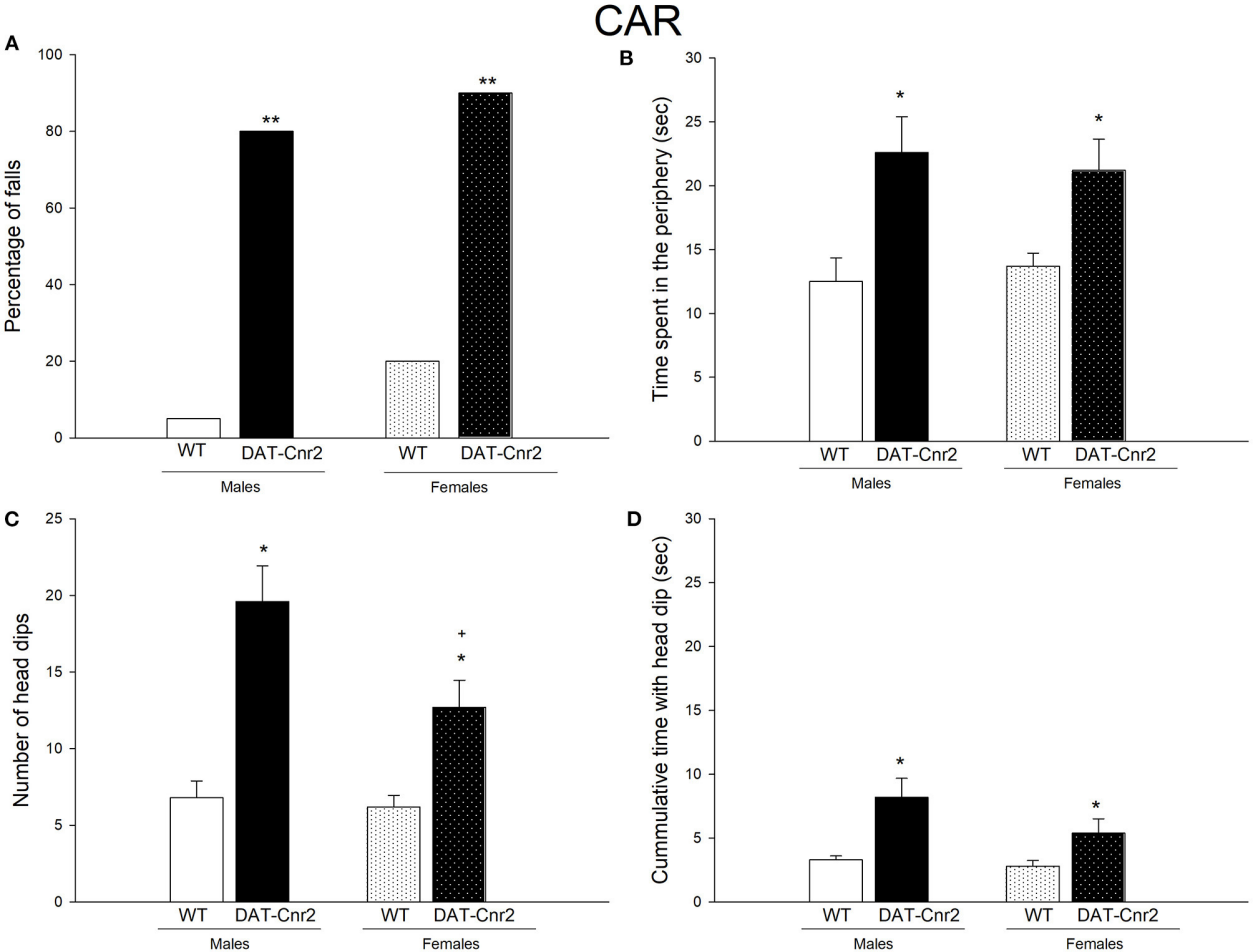
Performance in the Cliff avoidance reaction (CAR) test. **(A)** depicts the percentage of animals falling from the platform. **(B)** Time spent exploring the periphery of the elevated platform. **(C,D)** show the number of head dips and the accumulative time with the head dip. White and White with dots represent the WT mice and the Black and Black with dots represent DAT-Cnr2 mice. The clear bars are the males and the bars with dots represent the females. Data are expressed as mean ± SEM (n = 10 mice per group). **P < 0.01, Fisher F test for **(A)**. Two-way ANOVA followed by Tukey *p < 0.05 for genotypes; ^+^*p* < 0.05 for sex.

Figure 3A shows the percentage of DAT-*Cnr2* adolescent male and female mice falling from the platform. As it can be seen, among 80–90% of the animals fell. This percentage is significant (*p* < 0.001). This is important since male and females DAT-*Cnr2* adolescent mice showed an impairment in the cliff avoidance reaction (CAR). The factor genotype (WT and DAT-*Cnr2*) and the factor sex (male and female) were analyzed by a two-way ANOVA test for the rest of the parameters. The time spent exploring the periphery of the platform (Figure 3B), considered to be a risky behavior, is significantly higher by the male and female DAT-*Cnr2* mice [*F*_(1, 36)_ = 17.055, *p* < 0.001]. The number of head dips was significantly higher in DAT-*Cnr2* male and female adolescent mice [*F*_(1, 36)_ = 36.171, *p* < 0.001]. There was a difference in the factor sex in this parameter [*F*_(1, 36)_ = 5.445, *p* <0.025], the DAT-*Cnr2* female exhibited less head dips than the males of this genotype. Furthermore, the accumulated time spent with the head dipped is significantly higher in the DAT-*Cnr2* mice [*F*_(1, 36)_ = 15.171, *p* < 0.001], Figures 3C,D, respectively.

### Performance in the DDM and EDM T-Maze Test of the Male and Female Adolescent Mice

**Figure 4:**
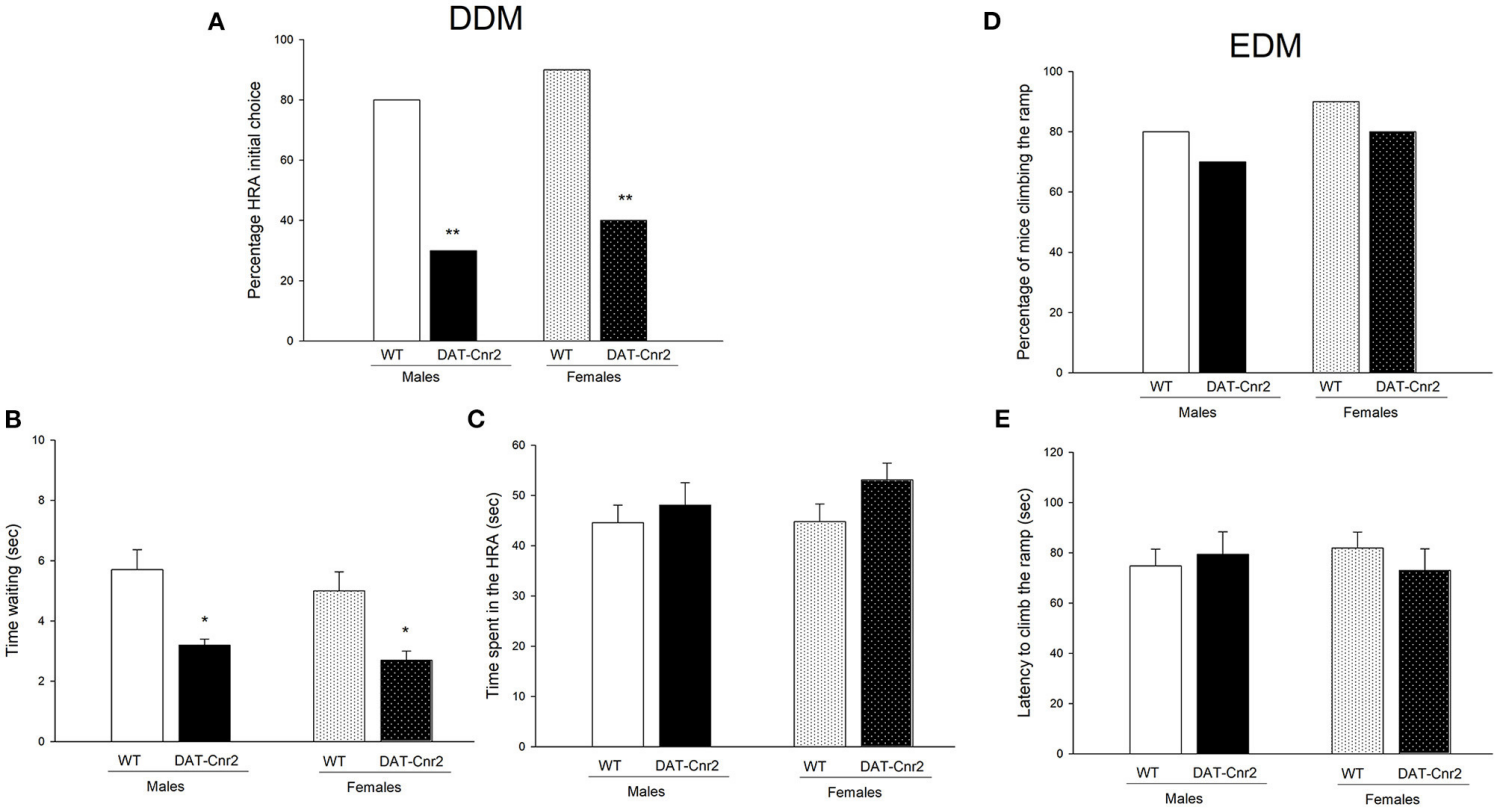
Performance in DDM and EDM. **(A)** shows the percentage of mice who chose the HRA as its first choice, exhibited in adolescent male and female mice **P < 0.01, Fisher F test for **(A)**. The time spent near by the barrier (i.e. waiting time) is shown in **(B)** and in **(C)**, the time spent in the HRA by the animals is shown. The white with dots represent the WT mice and the black and the black with dots represent DAT-Cnr2 mice. The clear bars are the males, and the bars with dots represent the females. Data are expressed as mean ± SEM (*n* = 10 mice per group). Two-way ANOVA followed by Tukey *p < 0.05 for genotypes. **(D)** shows the percentage of animals climbing to reach the HR in the EDM, and **(E)** exhibits the latency to climb the ramp to reach the HR.

As can be seen in Figure 4A, the proportion of DAT-*Cnr2* adolescent mice that chose the HRA is significantly reduced in comparison with the proportion exhibited by WT mice. These percentages remained similar among sexes, suggesting that DAT-*Cnr2* mice tended to choose the small but immediately available reward instead of the large delayed reward, which is a sign of impulsivity. Another sign of impulsivity was the time spent next to the barrier that blocked the HR. Two-way ANOVA revealed a significant difference [*F*_(1, 36)_ = 23.617, *p* < 0.001] for genotype factor. DAT-*Cnr2* adolescent mice spent significantly less time waiting for the HR (Figure 4B). Despite the first choice, the amount of time spent in the HRA was not different between the groups in the 3 minute period after they had the HR accessible (barrier removed with a delay of 10 s, and the animal was allowed to explore for 3 min after). Implication that the reward process was not altered (Figure 4C). There were no significant differences in the latency to climb the ramp in the EDM, WT and DAT-*Cnr2*, or in the percentage of animals climbing. As it can be seen in Figures 4D,E.

### Distance Traveled in Response to Doses of Amphetamine in Male and Female Adolescent Mice

**Figure 5:**
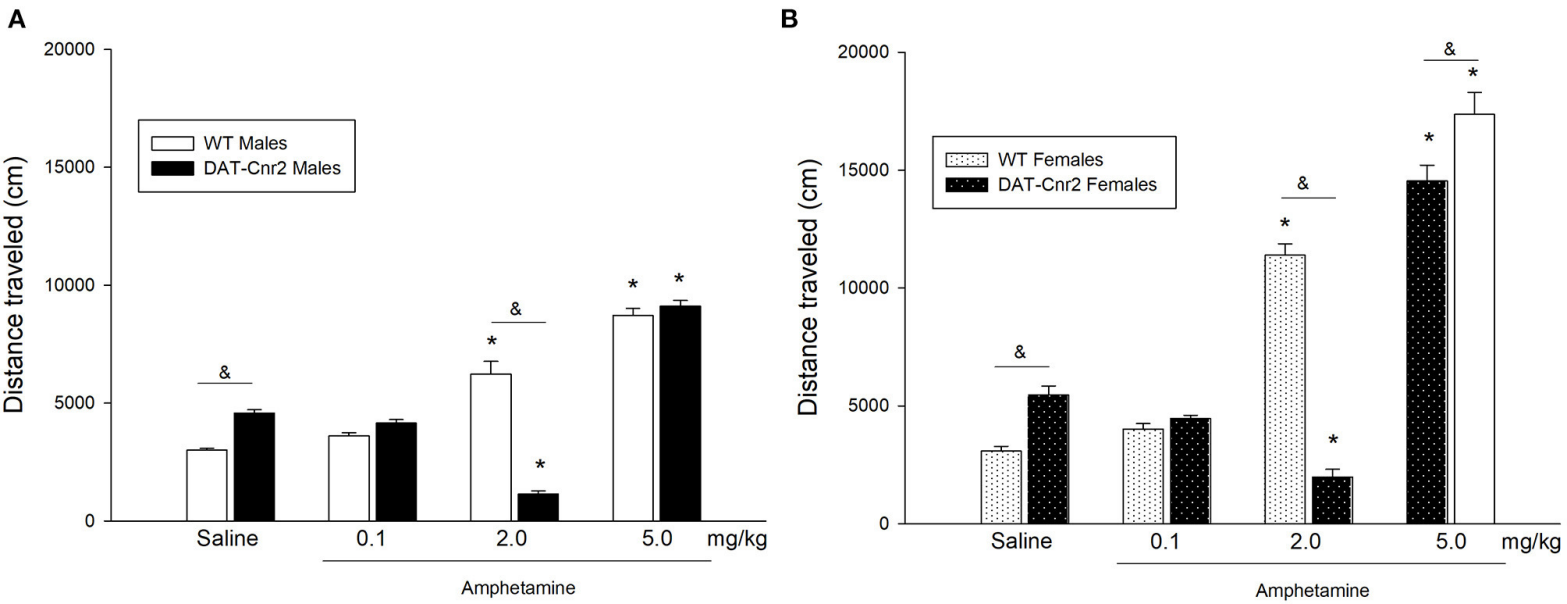
Locomotor activity in adolescent DAT-*Cnr2* cKO mice in response to different doses of amphetamine. Distance traveled in 30 min in the open field in response to 0.1, 2.0 and 5.0 mg/kg of amphetamine or its vehicle (saline). The white bars represent the WT and the black bars represent DAT-Cnr2 cKO mice. **(A)** males and in **(B)** females. Data are expressed as mean ± SEM (*n* = 10 mice per group). Three-way ANOVA followed by Tukey *post hoc* test *p < 0.05 for treatment and &*p* < 0.05 for genotype.

A three-way ANOVA was performed to analyze the effect of genotype (WT or DAT-*Cnr2*), sex (male or female) and treatment (0.1, 2.0, 5.0 mg/kg amphetamine or its vehicle) on the distance traveled in a 30 min duration session on the Open Field test. Figure 5 shows the mean number and SEM of the distance traveled in centimeters (cm) for each group. There was a significant 3-way interaction [*F*_(3, 144)_ = 13.50; *p* < 0.001]. There was also a 2-way interaction of Genotype and Treatment [*F*_(3, 144)_ = 123.43; *p* < 0.001] and another 2-way interaction of Sex and Treatment [*F*_(3, 144)_ = 63.76; *p* < 0.001]. Simple main effects analysis showed that genotype, sex and treatment had a statistically significant effect on the distance traveled [*F*_(1, 144)_ = 17.01; *p* < 0.001 for genotype; F_(1, 144)_ = 196.08; *p* < 0.001 for sex and F_(3, 144)_ = 426.72; *p* <0.001]. Tukey's *post hoc* test showed that in both male and female mice, the distance traveled was significantly higher than the one exhibited by the WT mice in response to saline. This result is related with the basal hyperactivity phenotype of DAT-*Cnr2* mice, and therefore was expected. The low dose of amphetamine (0.1 mg/kg) had no significant effect on the distance traveled in both genotypes nor in both sexes. The dose of 5 mg/kg increased the distance traveled in both WT and DAT-*Cnr2* mice, in male and female mice. Remarkably, the 2.0 mg/kg dose of amphetamine significantly changed the distance traveled in both genotypes, but in the opposite direction. In the male WT adolescent mice, 2.0 mg/kg of amphetamine induced an increase (3,000 ± 178 vs. 6,227 ± 154). However, in the DAT-*Cnr2* mice, the same dose induced a significant decrease (4,574 ± 142 vs. 1,141 ± 131, cm) in the distance traveled in a 30 min session. In WT female mice there was a significant increase (3,181 ± 194 vs. 11,391 ± 484) in the distance traveled, and in DAT-*Cnr2* female mice, there was a significant decrease (5,457 ± 385 vs. 1,978 ± 330).

### Effect of a Single Low Dose Administration of Amphetamine in the EPM Parameters in Adolescent Male and Female

**Figure 6:**
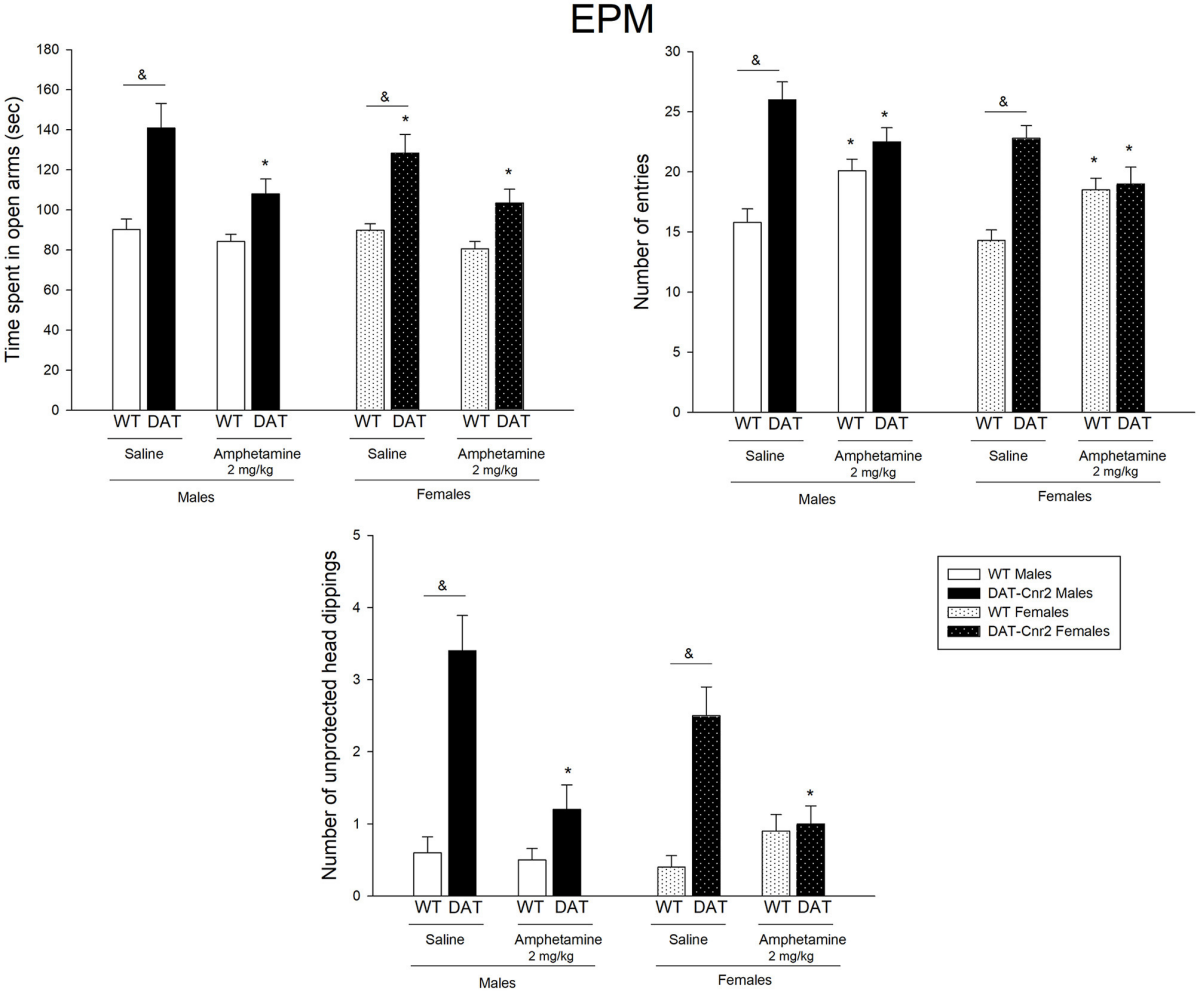
Performance of adolescent DAT-*Cnr2* male and female mice in response to a single administration of a low dose of amphetamine on the specific parameters of the EPM. Time spent in the open arms of the maze, expressed in seconds over 5 minutes, number of entries in total as general locomotor activity during the test and number of unprotected head dips (UHD) as a measure of reduced inhibition. Data are depicted mean ± SEM. The white bars represent the WT and the black bars represent DAT-*Cnr2* cKO mice. Males depicted in clean bars and females in bars with dots. Data are expressed as mean ± SEM (*n* = 10 mice per group). Three-way ANOVA followed by Tukey *post hoc* test *p < 0.05 for treatment and &*p* < 0.05 for genotype.

A three-way ANOVA was performed to analyze the effect of genotype (WT or DAT-*Cnr2*), sex (male or female) and treatment (2 mg/kg amphetamine or its vehicle) on the three parameters of the EPM: time spent in the open arms of the maze, number of entries to the arms and number unprotected head dips (UHD). Figure 6 shows the mean number and SEM of the parameters for each group. For the time spent in the open arms, there were no significant interactions among factors. Simple main effects analysis showed that the time spent in the open arms is different between genotypes [*F*_(1, 72)_ = 45.95, *p* < 0.001]. And a main effect of treatment was also found [*F*_(1, 72)_ = 13.23, *p* < 0.001]. Tukey's *post hoc* test showed that in both male and female DAT-*Cnr2* mice, the administration of saline, i.e. basal conditions, the time spent in the open arms displayed by the DAT-*Cnr2* mice was significantly high in comparison with the time spent by the WT (WT♂ = 90 ± 5 vs. DAT-*Cnr2*♂ = 140 ± 12 and WT♀ = 89 ± 3 vs. DAT-*Cnr2*♀ = 128 ± 9). The administration of 2.0 mg/kg of amphetamine did not modify the time spent in the open arms in the WT animals, but the same dose reduced it in the DAT-*Cnr2* mice of both sexes, reducing the difference observed in basal conditions. The time spent in open arms is considered a measure for anxiety-like behavior, so DAT-*Cnr2* mice displayed less anxiety-like behavior in the test. The next parameter analyzed, the number of entries in the EPM, there was no significant 3-way interaction. A 2-way interaction of Genotype and Treatment [*F*_(1, 72)_ = 23.67; *p* < 0.001] was found. Simple main effects analysis showed that genotype and sex had a statistically significant effect on the number of entries [*F*_(1, 72)_ = 44.23; *p* < 0.001 for genotype and [*F*_(1, 72)_ = 9.10; *p* = 0.004]. *Post hoc* analysis showed that the number of entries by DAT-*Cnr2* male and female mice was significantly higher than the number entries by the WT counterparts. The administration of a single low dose of amphetamine produced an increase in the number of entries by the WT (from 15 to 20 in males and from 14 to 18 in females), but produced a decrease in the DAT-*Cnr2* (from 26 to 22 in males and from 23 to 19 in females). The last parameter measured in this experiment was the number of unprotected head dips (UHD). This behavior consists of leaning the head out of the open arms of the maze. This is not a common parameter to be collected, but because the DAT-*Cnr2* mice display this behavior frequently, it was measured. There was no significant 3-way interaction. A 2-way interaction of Genotype and Treatment [*F*_(1, 72)_ = 37.67; *p* < 0.001] was found. The analysis of simple main effects showed that genotype [*F*_(1, 72)_ = 71.47; *p* < 0.001] and treatment [*F*_(1, 72)_ = 27.16; *p* < 0.001] had a statistically significant effect on the number of UHD. *Post hoc* analysis showed that the number of UHD was significantly higher in DAT-*Cnr2* mice, male and female, in comparison with the WT mice of both sexes. WT mice rarely present UHD, and in the DAT-*Cnr2* genotype, this parameter was augmented. In response to amphetamine (2 mg/kg), DAT-*Cnr2* mice displayed a reduction in the number of UHD and in the WT, the drug did not modify this parameter.

### Effect of a Single Low Dose Administration of Amphetamine in the Performance of the NOR Test in Adolescent Male and Female Mice

**Figure 7:**
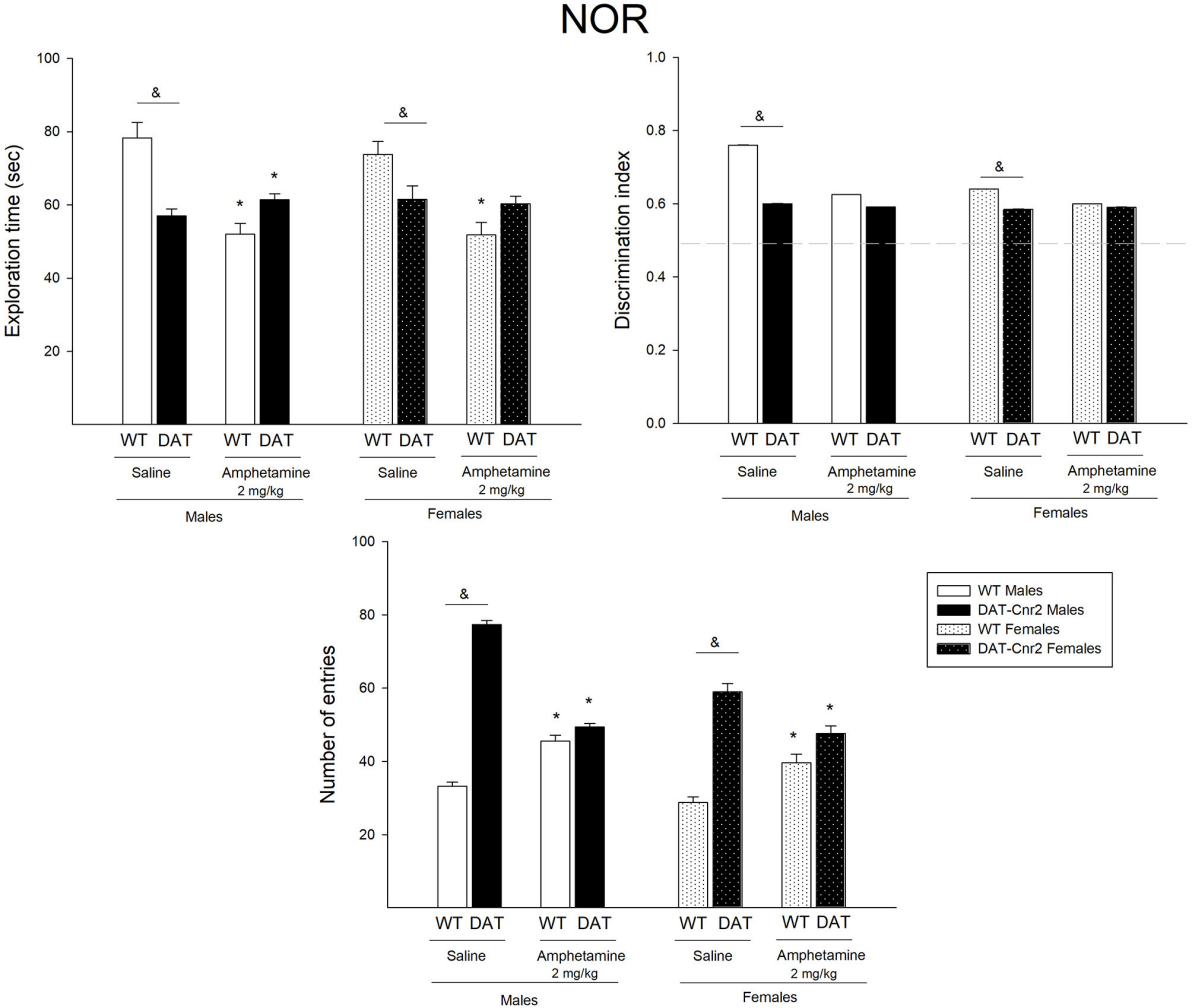
Performance of adolescent DAT-*Cnr2* male and female mice in response to a single administration of a low dose of amphetamine on the NOR test. The exploration time of DAT-*Cnr2* and WT mice. Discrimination index of both genotypes and on the total number of entries of both genotypes. Data are depicted as mean ± SEM. The white bars represent the WT and the black bars represent DAT-*Cnr2* cKO mice. Males depicted in clean bars and females in bars with dots. Data are expressed as mean ± SEM (*n* = 10 mice per group). Three-way ANOVA followed by Tukey *post hoc* test *p < 0.05 for treatment and &*p* < 0.05 for genotype.

A three-way analysis of ANOVA was performed to analyze the effect of genotype (WT or DAT-*Cnr2*), sex (male or female) and treatment [amphetamine (2 mg/kg) or saline] on the three parameters of the NOR: exploration time total, discrimination index and number of entries. Figure 7 shows the mean number and SEM of the parameters for each group on the performance of the mice in the NOR test.

For the exploration time, there were no significant interactions among factors, except for the genotype and treatment [*F*_(1, 72)_ = 35.30, *p* < 0.001]. Simple main effects analysis showed that the factor Genotype [*F*_(1, 72)_ = 3.28, *p* = 0.04] and the factor treatment [*F*_(1, 72)_ = 27.07, *p* < 0.001] had a statistically significant effect on the exploration time along the test. Tukey's *post hoc* test showed that in both male and female DAT-*Cnr2* mice, the exploration time was significantly lower than the one exhibited in the WT mice (WT♂ = 78 ± 4 vs. DAT-*Cnr2*♀ = 57 ± 2 and WT♀ = 73 ± 3 vs. DAT-*Cnr2*♀ = 61.5 ± 2). The administration of a single dose of amphetamine reduced the exploration time in the WT male mice and with a slight increase in the DAT-*Cnr2* mice. The same trend was found in female, but the difference was only significant in the WT female mice.

The discrimination index (DI) is a parameter for cognition. For this parameter, there was no significant 3-way interaction. A 2-way interaction of Genotype and Treatment [*F*_(1, 72)_ = 7.81; *p* = 0.007] was found. Simple main effect analysis showed that genotype and treatment had a statistically significant effect on the DI [*F*_(1, 72)_ = 22.02, *p* < 0.001 for genotype and *F*_(1, 72)_ = 7.54, *p* = 0.008]. *Post hoc* analysis showed that in basal conditions, the index was lower in the DAT-*Cnr2* male and female mice, but since all DI are higher than 0.5, both genotypes recognized the new object, indicating that there were no cognitive deficits. The administration of a single dose of amphetamine reduced the DI only in the WT male mice.

For the number of entries in the NOR test, there was a significant 3-way interaction [*F*_(1, 72)_ = 14.57; *p* < 0.001]. There was also a 2-way interaction between Genotype and Treatment [*F*_(1, 72)_ = 173.75; *p* < 0.001], between Genotype and Sex [*F*_(1, 72)_ = 4.36; *p* = 0.04] and between Treatment and Sex [*F*_(1, 72)_ = 10.14; *p* = 0.002]. Simple main effect analysis showed that genotype, sex and treatment had a statistically significant effect on the number of entries [*F*_(1, 72)_ = 331.28; *p* < 0.001 for genotype; *F*_(1, 72)_ = 11.81; *p* < 0.001 for treatment and *F*_(1, 72)_ = 41.37; *p* < 0.001 for sex]. Tukey's *post hoc* test showed that in both male and female mice, the number of entries were significantly higher in the DAT-*Cnr2* male and female mice in response to vehicle in comparison with the WT males (WT♂ = 33 vs. DAT-*Cnr2*♂ = 77 and WT♀ = 28 vs. DAT-*Cnr2*♀ = 59), and that the administration of a single low dose (2 mg/kg) of amphetamine induced an increase in the WT (45 in ♂ and 39 in ♀) and a decrease in the DAT-*Cnr2* mice (49 in ♂ and 47 in ♀).

### Cd11b Staining of the Hippocampal Dentate Gyrus and Cornu Ammonis (CA)

**Figure 8:**
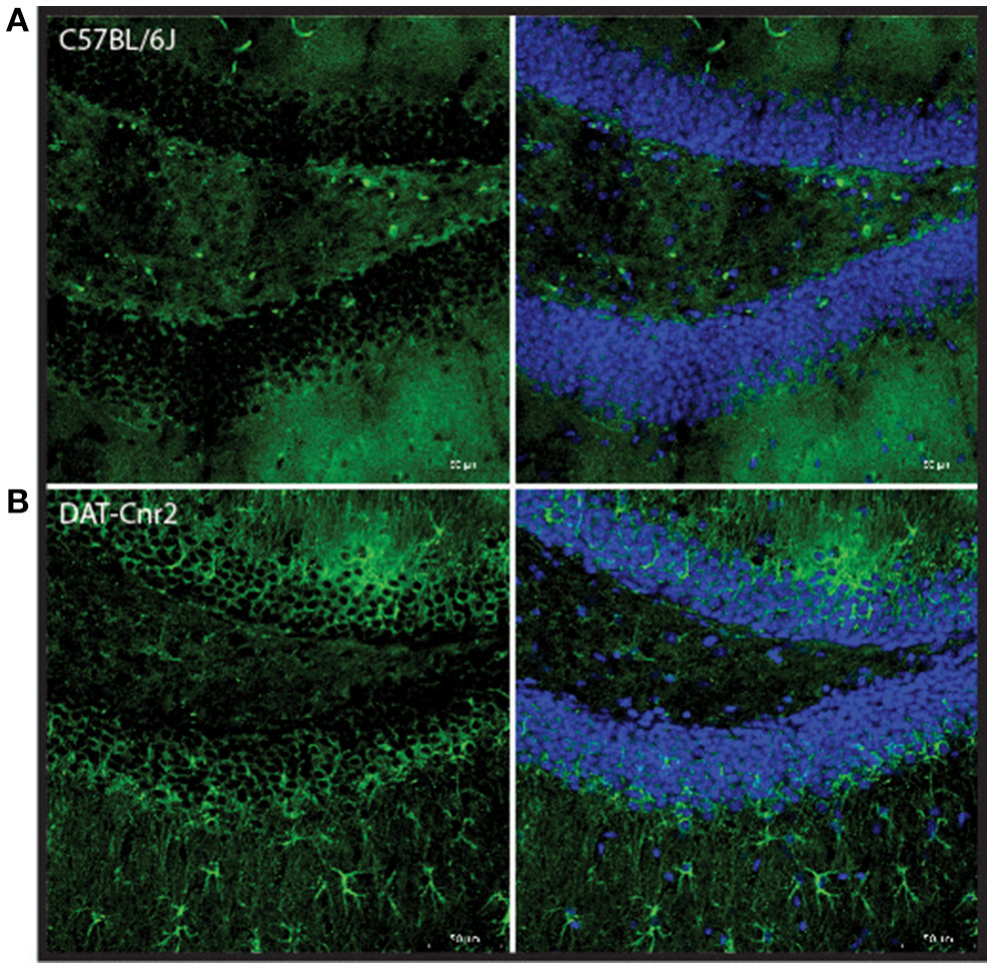
Representative images of Cd11b staining in dentate gyrus region of the hippocampus in male C57BL/6J **(A)** and DAT-*Cnr2* cKO **(B)** male mice. Cd11b immunofluorescence (green). Merged immunofluorescence of Cd11b and DAPI (blue).

The Hippocampus is a region of the brain associated with memory and cognitive function that contains DA neurons and other neurotransmitter systems. Coronal sections of dentate gyrus and cornu ammonis (CA) regions of the hippocampus from DAT-*Cnr2* and WT mice were stained with Cd11b, a marker for microglia activation, to investigate the neuro-immuno modulating effects of CB2Rs. Microglia activation detected by Cd11b was enhanced in the dentate gyrus and CA in DAT-*Cnr2* than in WT mice implicating neuro-immuno modulatory effects of CB2R, shown qualitatively in Figures 8A,B, respectively. The DAT-*Cnr2* cKO may be more susceptible to microglia activation because of the deletion of CB2R in dopamine neurons that are intact in the WT mice. Furthermore, in the hippocampus DA neurons projects to midbrain regions that we have analyzed previously demonstrating that CB2R and not CB1R are expressed in the VTA DA neurons (32). Therefore, we analyzed immune-reactivity in the hippocampus that has projects to midbrain DA neurons.

## Discussion

The main findings arising from the present study are: DAT-*Cnr2* cKO male and female adolescent mice are highly hyperactive. The persistent hyper-locomotor activity phenotype of the DAT-*Cnr2* mice were present in the behavioral tests used and they do not habituate to the environment with repeated exposure. DAT-*Cnr2* adolescent mice of both sexes exhibit high impulsive behavior. The administration of a single low dose of amphetamine induced a paradoxical effect characterized by a significant reduction of the locomotor activity response in the DAT-*Cnr2* mice, while producing a significant increase in the locomotor activity response of WT animals. This reduction in locomotor activity was extended to the other tests (EPM and NOR) in response to amphetamine treatment in the DAT-*Cnr2* mice. This treatment also narrowed the differences between genotypes in several parameters of EPM test and NOR task. We previously reported that DAT-*Cnr2* mice were hyperactive at adulthood (32). In the present study, we demonstrate that this hyperactive phenotype of the DAT-*Cnr2* mice is expressed from the early stages of development. This high increase in locomotor behavior was exhibited in other type of environments, like in the maze and the three-chambered arena (EPM and NOR), as well as in the arena of the OF test. Overall, the performance of the adolescent mice in both genotypes differs from their adult counterparts (data not shown). Adolescent rodents are in general more active and more impulsive, and they exhibit greater levels of novelty-seeking behavior and risk-taking relative to adults (45), highlighting the importance of testing the DAT-*Cnr2* mice during this particular developmental stage. This is because adolescence is a critical period for the neurobiological development of the brain (46), and because neurodevelopmental disorders such as attention-deficit hyperactivity disorder (ADHD) emerge during childhood and adolescence (47).

**Figure 9:**
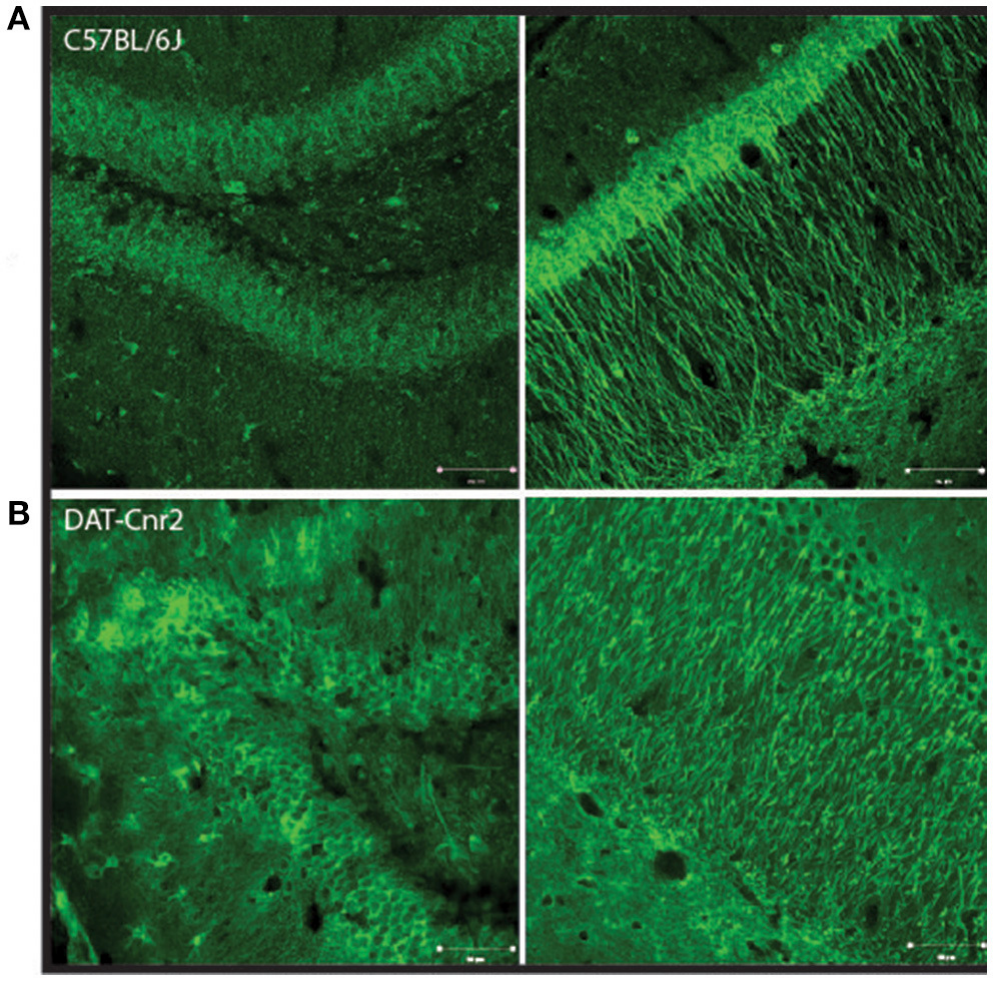
Representative images of Cd11b staining in dentate gyrus left and cornu amonis 1 (Right) regions of the hippocampus in male C57BL/6J **(A)** and DAT-*Cnr2* cKO **(B)** male mice. Cd11b immunofluorescence (green).

DAT-*Cnr2* cKO mice do not innately express CB2R on dopaminergic neurons in the midbrain. In the midbrain, CB2R are localized in the postsynaptic cell body of dopamine neurons (13). Thus, its activation hyperpolarizes the membrane's potential and inhibits postsynaptic neuronal function, i.e., reduces neuronal excitability through the CB2R-associated modulation of K+ channel function (48), suggesting that CB2R in midbrain modulate a variety of DA-associated functions and behaviors. If CB2R acts as a negative feedback regulator in the dopaminergic system, then with the deletion of CB2R in dopaminergic neurons, this particular modulation is no longer present. Since the deletion of the CB2R in dopamine neurons induces, among other characteristics, a hyperactive phenotype, CB2R must be involved in the regulation of locomotor activity. In support of this notion, it has been reported that the administration of a high dose of selective CB2R agonist (GW405833) causes ataxia and a loss of motor coordination (49). Therefore, the overstimulation of CB2R with a selective agonist induces ataxia, and its deletion from the dopamine neurons causes a hyperactive phenotype. One of the core effects of psychostimulants is the increase in locomotor activity. In DAT-*Cnr2* adolescent mice, consistent with the results in adult mice (34), the injection of a low dose (2 mg/kg) of amphetamine induced a reduction in distance traveled in the OF test, which is contrary to significant increase in distance traveled that was observed in WT animals. This effect is specific to this particular dosage, since the higher and the lower dosages that were tested induced no effects and an increase in the distance traveled, respectively. This also seems to be an effect provoked by this particular psychostimulant, since in OF tests; cocaine induced an increase in the distance traveled in the DAT-*Cnr2* as well as in the WT (34). In mice overexpressing brain CB2R, the administration of cocaine induced a decrease in locomotor responses to cocaine (50), suggesting that CB2R is important in the effects of locomotor activation induce by psychostimulants (Figures 9A,B).

Psychostimulants such as methylphenidate and amphetamine are the most important drugs prescribed to control ADHD symptoms (51, 52) and one of the most reliable evidence for the involvement of dopamine circuits in ADHD. The central action of psychostimulants is the facilitation of dopamine (and noradrenaline) transmission within the mesocorticolimbic system (31). The mesocortical system originates in the Ventral Tegmental Area (VTA) and projects to cortical areas, the prefrontal cortex (CPF) and the parietal and temporal cortex. These dopamine projections modulate circuits that play a role in executive functions, including motor control, behavioral inhibition, attention, and working memory (35, 53). The deficient dopamine-mediated modulation of PFC circuits (35) could be related with attention impairments and executive functions. This is because the mesolimbic system originates in the VTA and projects to limbic areas. This circuit is linked with the reward process (48). It has been suggested that deficiencies in reinforcement of appropriate behavior and/or deficient extinction of previously reinforced behavior could be related with ADHD symptoms like hyperactivity in a familiar environment, impulsiveness, deficiency in sustained attention, among others (35). These two systems arise from the VTA—the dopaminergic neurons of the VTA receive synaptic information from many brain areas including inputs directly from the prefrontal cortex and from other areas, as well as other indirect inputs (54). It is in the VTA where CB2R are knocked out, and therefore its modulation is not present in the DAT-*Cnr2* mice, indicating that in these animals the dopaminergic transmission is disturbed, highlighting the behavioral features of these animals. In the WT mice, with a normal regulation of the dopaminergic brain system, amphetamine induced hyperactivity by blocking dopamine reuptake and facilitating its release with an enhanced locomotor activity response (55). However, in the DAT-*Cnr2* mice, a highly hyperactive strain, the administration of a low single dose of this drug reduced that hyperactivity. Remarkably, the hyperactivity exhibited by people with ADHD is paradoxically reversed upon administration of amphetamine, meanwhile in healthy people, psychostimulants have a characteristic effect of increasing activity (56). Amphetamine also improves inhibitory control and cognition in people with ADHD (57). Previous studies have proposed that paradoxical effects of psychostimulants in ADHD do not simply imply a decrease in motor activity; rather, they appear to modulate motor activity through appropriate increase and decrease of dopamine influx on different brain areas. For example, cognitive functioning by the prefrontal cortex relies on optimal levels of mesocortical DA and is impaired either by reduced or excessive DA transmission (58, 59). Therefore, it is possible that ADHD symptoms are related to changes in dopaminergic transmission but also in other neuromodulatory systems that regulates them, among them the endocannabinoid system.

The present study initiates dialog on the use of the DAT-*Cnr2* cKO mice as a model of ADHD. ADHD is a neuropsychiatric disorder characterized by persistent pattern of inattention and/or hyperactivity-impulsivity that interferes or reduces the quality of social, academic, or occupational functioning in (DSM-5). It is an early-onset disorder, prevalent in both sexes that frequently persists into adulthood. The inattention component of ADHD is manifested as daydreaming, distractibility, and difficulty focusing on a single task for a prolonged period, whereas the hyperactivity component is expressed as fidgeting, excessive talking, and restlessness. While these symptoms alone can be highly disruptive, people with ADHD are also at increased risk of comorbidity, and later life disorders including addiction, eating disorders, anxiety and depression (60). The most widely used treatments are psychostimulants, most commonly methylphenidate (Ritalin) or a mixture of amphetamine salts (Adderall) (61). Animal models in psychiatry aim to model one or more of the core symptoms of the disorder that should meet some criteria for its validation. A good animal model of ADHD must meet three validation criteria (35): (1) Face validity—it must represent the behavioral characteristics of the human disorder; (2) Construct validity—conform to a theoretical rationale for the pathophysiology of the disorder; and (3) Predictive validity—be able to predict unknown aspects of the disorder, relating to behavior, genetics, neurobiology or treatment. In this case, the hyperactivity of the DAT-*Cnr2* mice, which is consistent and persistent since adolescence, could fulfill the face validity of ADHD. Since DAT-*Cnr2* mice responded with a reduction in locomotion, it could be considered representative of predictive validity. However, since the complete etiology and neurobiology is still a subject of investigation, it makes the validity more difficult to assess. We have noted the limitations that to be useful animal model does not have to be perfect replication of the disorder in terms of predictive, face and construct validity. In this study however, there is no doubt that dopamine is related. There are three subtypes of ADHD, defined by a combination of the three major symptoms (inattentive subtype, the hyperactive-impulsive subtype, and the combined subtype) (62). DAT-*Cnr2* mice seems closer to the hyperactive-impulsive subtype. The neurobiology of ADHD has strongly implicated the lateral prefrontal cortex, dorsal anterior cingulate cortex and striatum, integrated in the frontostriatal brain network that is related to regulation of behavior, and therefore its dysfunctional outcomes is a core symptom of ADHD. Dysregulation in catecholamine neurotransmission has been implicated in the pathophysiology of ADHD (63). Converging evidence suggests a primary role of disturbances in dopamine neurotransmission and this system has been most extensively studied (64, 65). The fact that psychostimulants such as amphetamine and methylphenidate, the most common first-line treatments for ADHD, enhance extracellular dopamine, suggests that an underlying dopamine deficit is corrected by these drug (56). It has been proposed that underlying ADHD are impairments in dopaminergic receptor function (66, 67) or polymorphisms in dopaminergic receptors (68) or in the dopamine transporters (DAT) (69, 70). Furthermore, dopamine transporter knockout mice exhibit maladaptive impulsive rodent behavior evaluated in the CAR test (40).

In studies of the neurobiology of ADHD, both the dopaminergic system and the endocannabinoid system is involved in the regulation of dopaminergic neurotransmission. There is some evidence available about the implication of the endocannabinoid system components in ADHD, although they are related with CB1R that are known to form dimers with CB2R. Human studies have reported that there are SNP (Single nucleotide polymorphisms) variants at the CNR1 gene, the gene that encodes for CB1R in an ADHD adolescent sample (71). However, the possible role of CB2R in ADHD has not been well-characterized. CB2R has been associated with other psychiatric disorders, such as schizophrenia (72, 73), depression (15), and bipolar disorder (74), among others. Regarding the other core symptom of ADHD: impulsivity, we used a specific test to evaluate impulsive-like behavior. First we observed that DAT-*Cnr2* mice exhibit an unusual number of head dips on the EPM, that is, they looked out into open arms of the maze that is elevated i.e., downward movement of mice' head toward the floor from the open arms. This parameter, although not frequently reported constituted a valid ethological parameter, considered “risk assessment” (75) since the animal instinctively should stay away from potentially dangerous events, in this case falling from the maze. Adolescence is a period of development characterized by impulsive and risk-seeking behaviors (76). However, DAT-*Cnr2* adolescent mice demonstrated head dips considerably more often than the WT. Impulsivity is a complex construct that describes a set of behaviors characterized by relative dominance of spontaneity. Examples include a preference toward obtaining immediate gratification over a delayed (yet ultimately more profitable) outcome, making “snap decisions” before evaluating available information, or aborting an initiated motor response (77), that can be dangerous. When impulsivity does not imply a decision-making component, i.e., choice, it can be defined as impulsive action (78). This observation encouraged our evaluation of the CAR for impulsive action and DDM and EDM for impulsive choices in this study.

Here we report that the DAT-*Cnr2* mice exhibited unprotected head dips and CAR impairment compared to WT mice, suggesting that DAT-*Cnr2* showed a reduction in the inhibition, this inability to withhold a response is a form of impulsive behavior, impulsivity of action (78). Another form of impulsivity is the impulsive choice, defined as behavior without foresight (78); it is frequently evaluated by delay discount defined by the choice for a small, immediate reinforcer over a larger, delayed reinforcer. In this study, we demonstrate that DAT-*Cnr2* mice exhibit a deficient response in the delay decision making. Supporting the notion that these animals are impulsive on two particular measure of impulsivity used (impulsive choice vs. impulsive action). A previous study demonstrated that CB2R mediated regulation of impulsive-like behavior (79) in agreement with our current findings. In another study using the SHR rats, a model reproducing some features of ADHD, the administration of the cannabinoid WIN55212-2 modulated impulsive behavior, tested in a delay reinforcement task (80). Although the effect was considered to be mediated by CB1R, WIN55212-2 has affinity for both CBRs. The authors concluded that the antagonism of cannabinoid receptors might be effective in reducing impulsive symptoms present in ADHD, since WIN55212-2 decreased the choices of the large reward, suggesting that CB1R plays a relevant role in impulsive behavior. The EPM test is based on conflict test in rodents, a desire to explore the surroundings and a fear of high and open places. Adolescent mice usually explore the open arms more than adults (81). Anxiety is a normal and important emotion—it evolves to alert the organism if there is a threat. Being afraid of high and open places is a survival trait, so an increase in the time spent in the open arms can be interpreted as a reduction of anxiety that is not adaptive. It has been reported previously that amphetamine at the dose tested (2 mg/kg) has no significant effect in WT mice (82). CB2R were originally believed to be predominantly expressed in immune cells and earlier studies were on the CB1Rs and the neuronal function of CB2R has been less investigated for CNS functions. While the mechanism(s) of CB2R have been controversial, the discovery of functional neuronal CB2R (14, 15, 83–85) and reports of enhanced CB2R during inflammation has raised questions regarding their roles in regulating neuroinflammation and behavior. The hippocampus is a brain region associated with memory and cognitive function and contains dopaminergic neurons and other modulatory neurotransmitter systems that are involved in the regulation of the hippocampal physiological state, and it has been demonstrated that CB2R play a modulatory role in this brain area (86, 87). Data from the present work reports that the Cd11b detected an enhanced microglia activation in the hippocampal dentate gyrus and CA in DAT-*Cnr2* than in WT mice implicating neuro-immuno modulatory effects of CB2R. Microglia cells play a role in many important neurodevelopmental processes, including synaptic pruning (88, 89). Microglial activation have been implicated in the pathophysiology of ADHD (90). This result provides additional evidence of the possible implication of microglia activation in the behavioral features observed in this mouse model and, future experiments should explore the involvement of other brain regions such as the midbrain and the prefrontal cortex.

In conclusion, the present results presents a putative animal model that was generated using *Cnr2*-LoxP targeting strategy to delete CB2R from dopamine neurons in the DAT-*Cnr2* cKO mice to study Attention Deficit Hyperactivity Disorder (ADHD). The animals have been validated using RNAscope *in-situ* hybridization with CB2R mRNA and TH probes for the cell-type specific deletion of CB2R from DA neurons (32). A remarkable feature is the paradoxical effects of amphetamine in reducing the exaggerated hyperactive phenotype in the DAT-*Cnr2* cKO mice, similar to the paradoxical clinical use of amphetamine containing compounds in the treatment of ADHD patients. The DAT-*Cnr2* cKO may be more susceptible to microglia activation because of the deletion of CB2R in dopamine neurons that are intact in the WT mice. Further studies and additional characterization are needed to compare the DAT-*Cnr2* cKO and other cell-type CB2R cKO mice that is ongoing. The results demonstrates DAT-*Cnr2* cKO mice with cell-type specific deletion of CB2R in midbrain dopaminergic neurons may represent a possible model for studying the neurobiological basis of ADHD.

## Data Availability Statement

The original contributions presented in the study are included in the article/Supplementary Material, further inquiries can be directed to the corresponding author/s.

## Ethics Statement

The animal study was reviewed and approved by IACUC. William Paterson University, Wayne, NJ 07470 USA.

## Author Contributions

AC-A and EO planned the experiments. Q-RL designed the constructs to generate DAT-Cnr2 cKO mice. AC-A, BS, MH, and RB conducted the behavioral experiments. MM and RB ran the NOR task. BS performed the IHC. AC-A wrote the manuscript and reviewed by Q-RL and EO. All authors contributed to the article and approved the submitted version.

## Funding

This work was supported by NIAAA-NIH grant AA027909. Neuroimmune behavioral effects of CB2 cannabinoid receptors to EO. Dean of College of Science and Health at William Paterson University—Dr. Venkat Sharma provides support for the Animal Facility and Student workers.

## Conflict of Interest

The authors declare that the research was conducted in the absence of any commercial or financial relationships that could be construed as a potential conflict of interest.

## Publisher's Note

All claims expressed in this article are solely those of the authors and do not necessarily represent those of their affiliated organizations, or those of the publisher, the editors and the reviewers. Any product that may be evaluated in this article, or claim that may be made by its manufacturer, is not guaranteed or endorsed by the publisher.
