# Supplementary material for: Cell-Type Specific Deletion of CB2 Cannabinoid Receptors in Dopamine Neurons Induced Hyperactivity Phenotype: Possible Relevance to Attention-Deficit Hyperactivity Disorder
Source: Front Psychiatry. 2022 Feb 8;12:803394. doi: 10.3389/fpsyt.2021.803394 (PMC8860836; doi:10.3389/fpsyt.2021.803394)
Supplement: Supplementary file 1 [file Table_1.DOCX]

**Supplemental data**

| **Sex** | **Males** | | **Females** | |
| --- | --- | --- | --- | --- |
| **Genotype** | **WT** | **DAT-*Cnr2*** | **WT** | **DAT-*Cnr2*** |
| Parameter/Test/Treatment | mean±SEM | mean±SEM | mean±SEM | mean±SEM |
| Distance traveled Session 1 | 3545.5±236.4 | 4626.6±158.9 | 3133.9±168.9 | 4181.9±138.4 |
| Distance traveled Session 2 | 2205.9±112.2 | 4276.5±223.6 | 2469.5±107.7 | 3834.0±143.2 |
| Distance traveled Session 3 | 2049.9±85.3 | 4251.5±211.6 | 2037.8±60.6 | 3557.3±151.1 |
| Distance traveled Saline | 3000.3±178.5 | 4574.8±142.1 | 3181.7±194.6 | 5457.2±385.4 |
| Distance traveled  0.1 mg/kg | 3608.7±129.2 | 4150.2±146.2 | 4011.5±236.3 | 4461.9±141.2 |
| Distance traveled  2 mg/kg | 6227.0±540.8 | 1141.0±131.7 | 11391.1±484.1 | 1978.8±330.9 |
| Distance traveled  5 mg/kg | 8710.5±309.7 | 9100.5±252.4 | 14542.7±666.1 | 17364.7±925.5 |
| Exploration Time Total | 90.2± 5.1 | 140.9±12.2 | 89.8±3.2 | 128.4±9.3 |
| Exploration Time Total + 2 mg/kg Amphetamine | 84.2±3.6 | 108±7.3 | 80.6±3.6 | 103.5±6.8 |
| Entries Total | 15.8±1.1 | 26±1.4 | 14.3±0.8 | 22.8±1 |
| Entries Total + 2 mg/kg Amphetamine | 20.1±0.9 | 22.5±1.1 | 18.5±0.9 | 19±1.3 |
| Unprotected Head Dips | 0.6±0.2 | 4.2±0.4 | 0.4±0.1 | 1.3±0.9 |
| Unprotected Head Dips + 2 mg/kg Amphetamine | 0.5±0.1 | 1.4±0.3 | 0.9±0.2 | 1±0.2 |
| Exploration time | 78.3±4.1 | 57±1.9 | 73.8±3.5 | 61.5±3.7 |
| Exploration time plus 2 mg/kg Amphetamine | 52±2.9 | 61.4±1.6 | 51.8±3.4 | 60.3±2 |
| Discrimination index | 0.697±0.01 | 0.603±0.001 | 0.645±0.02 | 0.585±0.01 |
| Discrimination index + 2 mg/kg Amphetamine | 0.625±0.01 | 0.591±0.001 | 0.603±0.01 | 0.598±0.001 |
| Total of Entries | 33.2±1.1 | 77.4±1.0 | 28.8±1.0 | 59±2.2 |
| Total of Entries + 2 mg/kg Amphetamine | 45.5±1.6 | 49.4±0.9 | 39.6±2.4 | 47.6±2.0 |
